# Exploring the Role of Perfusion in Skin Graft Viability on the Scalp and Lower Limb: An Analysis of Graft Bed, Margin, and Donor Skin Using Laser Speckle

**DOI:** 10.3390/jcm13247671

**Published:** 2024-12-16

**Authors:** André Pinho, Ana Brinca, Ricardo Vieira

**Affiliations:** 1 Dermatology Department, Hospitais da Universidade de Coimbra, Unidade Local de Saúde de Coimbra, 3004-561 Coimbra, Portugal; 2 Dermatology Clinic, Faculty of Medicine of the University of Coimbra, 3000-548 Coimbra, Portugal

**Keywords:** graft survival, time factors, laser speckle contrast imaging, skin transplantation

## Abstract

**Background/Objectives**: Reperfusion is a major determinant of skin graft viability. The contributions of the perfusion status of the wound bed, wound margin, and donor skin to the success of the skin graft are unclear. We aimed to evaluate the relationship between perfusion variables and graft necrosis extension on the scalp and lower limb. **Methods**: A prospective study was conducted on adults undergoing skin graft closure after skin cancer excision on the scalp (*n* = 22) and lower limb (*n* = 20). Perfusion was measured intraoperatively and non-invasively with laser speckle contrast imaging on the graft bed, margin, and donor skin. By day 28, graft necrosis extension was quantified. **Results**: On the scalp and lower limb, graft bed perfusion very strongly correlated with necrosis extension (r = −0.82, *p* < 0.001 and r = −0.94, *p* < 0.001, respectively). A significant correlation (r = −0.57, *p* = 0.01) between margin perfusion and necrosis extension was only observed on the lower limb. The donor skin perfusion and necrosis extension did not correlate in either location (*p* > 0.05). The graft bed perfusion explained 68% and 89% of the variation in necrosis extension on the scalp and lower limb, respectively. Regression models of necrosis extension based on graft bed perfusion were obtained. For each unit increase in the perfusion of the graft bed, a similar decrease in necrosis extension was observed on the scalp and lower limb (40 and 48 percentage points, respectively). **Conclusions**: Unlike the perfusion of the wound margin and donor skin, wound bed perfusion plays a significant role in skin graft viability and can predict necrosis extension.

## 1. Introduction

In dermato-oncologic reconstructive surgery, the success of a skin graft depends mainly on how quickly and effectively the vascular perfusion is reestablished in this tissue deprived of its original bed [1]. Skin grafts are technically more straightforward to perform than skin flaps, allowing for the coverage of small- to large-sized defects in areas without skin excess, and can be valuable in elderly patients with a poor functional status [2]. Still, skin grafts on the scalp or lower limb are especially prone to necrosis, with negative functional, cosmetic, emotional, and financial impacts. The reported graft failure rates range from 8% to 22% on the former and 34% to 66% on the latter location [3,4].

Under equivalent clinical and technical conditions, at least three perfusion-related factors are believed to play a pivotal role in the fate of a skin graft, and they are as follows: (1) the blood supply of the donor skin, (2) the perfusion of the wound bed, and (3) the vascularisation of the skin surrounding the defect [5,6,7]. Grafts harvested from highly vascularized donor sites are expected to heal better than those from poorly perfused regions. The native vascular network might serve as nonviable conduits through which the endothelium of the ingrowing vessels progresses [5]. A higher density of the native vascular network can thus enhance revascularisation. This rationale has been applied to new innovatively engineered extracellular matrices with residual natural vascular structures to facilitate angioconduction [8]. Grafts placed on a poorly vascularised bed are ischemic for a longer period and are more prone to necrosis than those placed on beds with a good blood supply [6]. Wounds with exposed bone, tendon, or cartilage deprived of intact periosteum, paratenon, or perichondrium, respectively, do not support a skin graft [9]. These sites should be allowed to granulate before the placement of a skin graft. Still, non-immediate closure does not necessarily guarantee the absence of late graft necrosis [10]. Moreover, skin grafts on the scalp, placed over intact aponeurotic galea, may have distinct outcomes, which could be attributable to differences in the wound bed perfusion [11]. The graft margin might contribute to graft survival by enhancing plasmatic imbibition or revascularisation, along with the graft bed [12]. Reperfusion from the wound margins and the wound bed has been documented in murine models of skin grafts [7]. Dermal substitutes are also revascularized from the marginal skin when applied over tissues exhibiting slow metabolism (bradytrohpic) [13].

Laser speckle contrast imaging (LSCI) is a non-invasive optical technique for measuring and visualising blood perfusion in biological tissues, including the skin, with good spatiotemporal resolution [14]. This method is based on analysing the backscattered speckle patterns produced by the interaction of coherent light, like a laser in the near-infrared spectrum, with moving red blood cells and stationary tissue [15]. For a wavelength of 780 nm, the penetration depth is around 300 μm [16]. This penetration depth is lower than that of laser Doppler imaging (around 1.0 to 1.5 mm), but unlike LDI, LSCI perfusion measurements are obtained much faster, almost in real time [16]. Additionally, LSCI does not require dye injection, avoiding the pharmacokinetic constraints associated with fluorescence angiography using indocyanine green [17]. Optical coherence tomography offers a higher spatial resolution than LSCI, enabling the detailed visualisation of the tissue microstructure. However, its limited field of view (less than 1.4 cm^2^) restricts the assessment of larger areas, which is a key advantage of LSCI [18]. So, LSCI is a valuable tool for intraoperative perfusion studies. It has been employed in the study of skin graft and skin flap perfusion in distinct fields such as dermato-oncology, oculoplastic, and burn surgery [19,20]. Its applications are broader and include neurosurgery, abdominal surgery, as well as in several medical conditions associated with endothelial vascular dysfunction [21]. Despite the increasing knowledge about the characterisation of skin (graft) perfusion with LSCI, most of it is purely academic and without direct clinical application. 

In previous research using LSCI, our group identified that scalp and lower limb skin grafts share the same reperfusion pattern. It takes 21 days for skin graft perfusion to surpass control skin perfusion in both locations, with the centre is better perfused than the periphery during the first three weeks [22]. Still, it remains unknown which of the three previously mentioned perfusion-related factors (donor skin, wound bed, or margin) influence skin graft viability more or how their contribution varies by anatomic location. If we could demonstrate that any of these factors significantly correlate with necrosis extension, its perfusion could be assessed intraoperatively to identify the patients with an increased risk of graft necrosis, according to which the procedure might be counter-indicated.

We aimed to evaluate the relationship between the perfusion (assessed on the donor skin, graft bed, or margin) and skin graft necrosis extension on the scalp and lower limb.

Our null hypothesis (H_0_) is that perfusion does not significantly correlate with skin graft necrosis extension on the scalp or lower limb. The alternative hypothesis (H_1_) is that perfusion and necrosis extension are significantly correlated.

## 2. Materials and Methods

### 2.1. Study Design and Population

We conducted an observational longitudinal prospective study with a 28-day follow-up at the Dermatology Department of the Unidade Local de Saúde de Coimbra in Portugal. The study followed the ethical standards outlined in the Declaration of Helsinki and the local ethics committee approved it.

All adult individuals referred to our department for skin cancer excision on the scalp or lower limb and suitable for skin graft closure were invited to be included in the study (i.e., a complete sampling). The inclusion criteria were age ≥ 18 years old, skin graft closure on the scalp or lower limb following skin cancer excision, ability to attend weekly follow-up visits for graft evaluation over four weeks, and capacity to provide informed consent. Exclusion criteria were established to minimise artefacts that could bias perfusion measurements, specifically, body temperature above 37.5 °C and the inability of the patient to remain still during LSCI recording. Additionally, patients with grafts with post-operative bacterial graft infection or haematoma were also excluded. Participants were divided into two groups based on the anatomical location of the defect, scalp and lower limb. Informed consent was obtained from every subject.

Recruitment occurred during the routine pre-operative evaluation between April 2023 and January 2024. Accepting an alpha risk of 0.05 and a beta risk of 0.20, the sample size for a correlation coefficient of ≥0.60 (strong or very strong correlation) was 20 participants per group.

### 2.2. Study Procedures

The procedures followed in this study were specifically designed to address the research objectives and were based on established principles of perfusion assessment and skin graft evaluation, as demonstrated in our previous pilot studies [11,23]. These studies provided valuable insights into the feasibility and reliability of using LSCI to evaluate skin graft perfusion, ensuring the scientific rigour of the current protocol.

The participants’ eligibility and the characteristics for analysis, such as age, sex, presence of arterial hypertension, dyslipidaemia, diabetes, current smoking status, and whether they were taking anticoagulant or antiplatelet drugs, were assessed in the pre-operative evaluation.

No change was made to our department’s reconstructive algorithms or postoperative wound care for this study. All surgeries were led by one of two surgeons with extensive experience in skin grafting. Lesions were removed after infiltration with lidocaine (2%) and epinephrine (1:100,000). Full-thickness skin grafts were harvested from the infraclavicular region for scalp defects. Split-thickness skin grafts with a 0.40 mm thickness were harvested from the anterior thigh for lower limb defects. Tie-over dressing was applied for one week, and a new dressing with petrolatum gauze was used afterwards.

**Figure 1:**
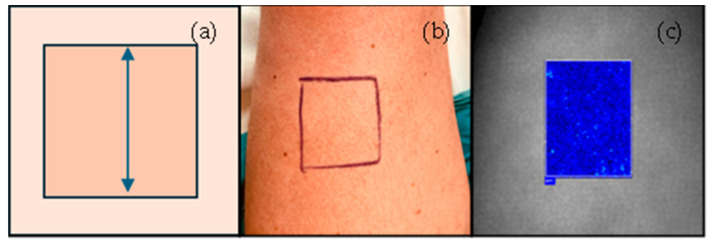
Schematic representation of the donor skin area (arrow) of a split-thickness skin graft for lower limb defect closure (**a**). Clinical image (**b**) and LSCI perfusion image (**c**) of donor skin. LSCI perfusion images range from black (no perfusion) to red (intense perfusion). LSCI also gives perfusion in arbitrary units (not presented) that can be normalised to the mean arterial pressure, yielding the cutaneous vascular conductance.

On day 0, the donor skin was delimitated with a skin marker, and perfusion was measured for 60 s (our “time of interest”) before local anaesthesia infiltration (Figure 1).

**Figure 2:**
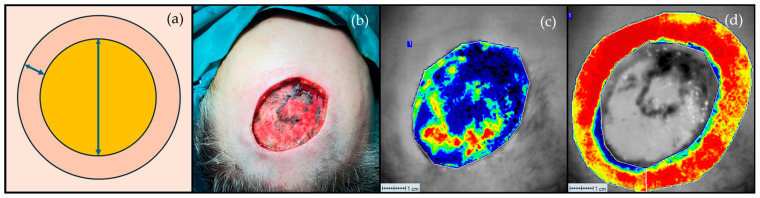
Schematic representation of the regions of interest relating to the graft bed (long arrow) and graft margin with a 1 cm width (short arrow) (**a**). Clinical image of the surgical defect (**b**), and LSCI perfusion image of the graft bed (**c**) and graft margin (**d**). Darker colours (black and blue) denote a poor perfusion assessed using LSCI, while the yellow to red colours represent a high perfusion.

Fifteen minutes after skin cancer removal, the perfusions of the graft bed and graft margin (defined as the skin surrounding the defect with a 1 cm width) were assessed intraoperatively for 60 s (Figure 2).

The leading surgeon performed these perfusion measurements with the PeriCam PSI NR^®^ LSCI system (Perimed, Järfälla, Sweden). Image analysis was undertaken using the manufacturer’s software (PimSoft 1.5.4.8078^®^, Perimed, Järfälla, Sweden). In each perfusion recording, “regions of interest” were manually defined as follows: the donor skin, the graft margin, and the graft bed. The software produces blood perfusion images of the scanned area in a semiquantitative colour scale, from black (meaning no perfusion) to red (intense perfusion). More importantly, LSCI provides quantitative perfusion values as arbitrary perfusion units (APU). For this study, the mean perfusion value of each “region of interest” during the 60-second “time of interest” was considered for statistical analysis. To better compare perfusion values between patients, the mean perfusion in APU was normalised to each participant’s mean arterial pressure (MAP), giving us the cutaneous vascular conductance (CVC), our reference of perfusion measurement. To avoid movement artefacts, the studied area was immobilised with a vacuum pillow (AB Germa™, Kristianstad, Sweden). 

**Figure 3:**
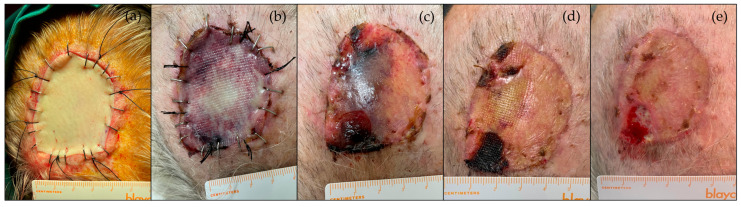
Evolution of a full-thickness graft healing on the scalp. Graft pictures were collected on days 0 (**a**), 7 (**b**), 14 (**c**), 21 (**d**), and 28 (**e**). From the clinical pictures of day 28, the necrosis extension was calculated with the software SketchAndCalc™.

All skin grafts were evaluated weekly for at least four weeks, and clinical photographs were collected (Figure 3). The necrosis extension was calculated from the pictures taken on day 28 by dividing the necrosis area by the graft area. These areas were delimitated with the software SketchAndCalc™ (version 6.3.0) by an independent investigator who did not participate in the surgeries or the post-operative follow-up. Necrosis was defined as any loss of surface integrity on day 28 (from epidermolysis that progressed to scab to full-thickness necrosis).

### 2.3. Statistical Analysis

Descriptive statistics for categorical variables are reported as counts and percentages. Continuous variables are presented as the means and standard deviation (SD), if they are normally distributed, or the median and interquartile range, if they are not normally distributed. A comparative analysis according to the anatomic location of the graft (scalp or lower limb) was performed using Chi-square, Fisher exact, independent-sample, and Mann–Whitney tests. A correlation analysis between perfusion and necrosis extension was performed, and linear regression models were obtained to identify statistically significant predictors of necrosis. All analyses were conducted using IBM SPSS Statistics^®^ (version 26), with a significance level set at <0.05. 

## 3. Results

A total of 42 skin grafts from 42 participants were included, 22 on the scalp and 20 on the lower limb. The characteristics of participants, defects, and grafts according to the anatomic location are represented in Table 1.

The median age of participants was 79 and 82 years, respectively, on the scalp and lower limb (*p* = 0.58). In both groups, most of the participants suffered from arterial hypertension and dyslipidaemia, and more than one-fifth had diabetes. The proportion of smokers was negligible. Regarding the characteristics of the participants, we only observed sex-related differences among the two groups. The proportion of women that underwent skin graft on the scalp (18%) was significantly lower than the proportion that underwent skin graft on the lower limb (70%) (*p* < 0.001).

The defect size did not differ significantly between groups, 11 cm^2^ in the scalp and 14 cm^2^ in the lower limb (*p* = 0.09). There was a tendency for a higher necrosis extension in the lower limb grafts (31%) than in the scalp (12%) (*p* = 0.06).

Regarding perfusion measurements, there were significant differences between anatomical locations (Table 2). The donor skin perfusion in the scalp group (1.4 APU/mmHg) was significantly higher than in the lower limb group (0.7 APU/mmHg) (*p* < 0.001). Graft margin perfusion on the scalp was more than double that observed on the lower limb (1.1 APU/mmHg and 0.51 APU/mmHg, *p* < 0.001). Graft bed perfusion did not differ significantly between the two locations (*p* = 0.69).

### Correlation Between Perfusion and Necrosis Extension

**Figure 4:**
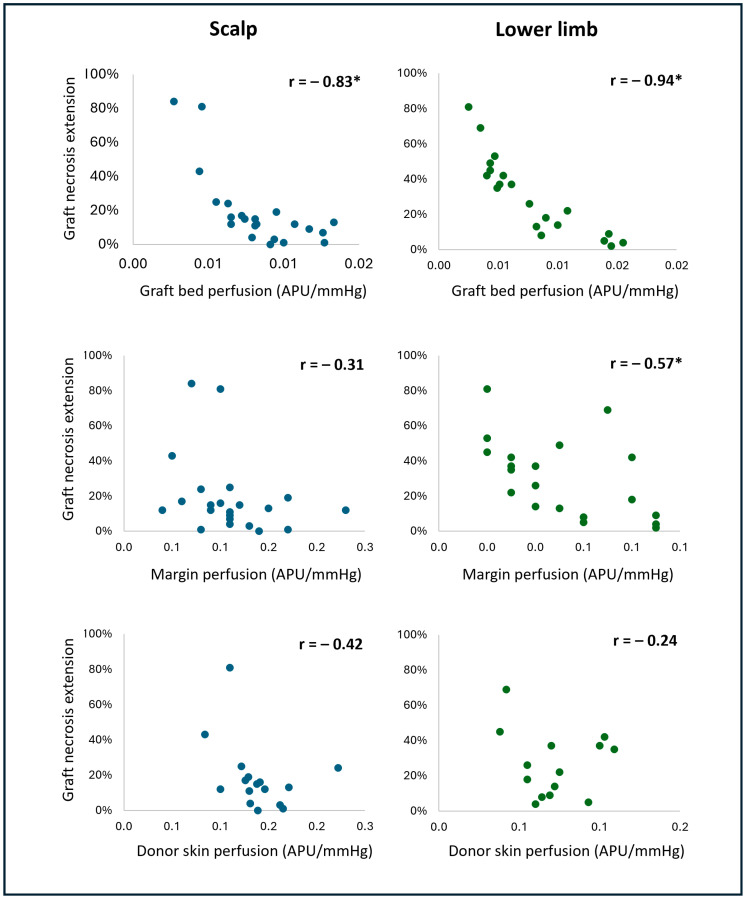
Scatter plots of perfusion, measured as cutaneous vascular conductance (CVC) [in arbitrary perfusion units (APU)/mmHg], on the graft bed, margin, and donor skin and necrosis extension on day 28, either on the scalp or lower limb. Statistically significant correlations (r) are denoted by an asterisk (*).

Figure 4 presents scatter plots depicting the distribution of necrosis extension on day 28 and the perfusion (as CVC, in APU/mmHg) assessed intraoperatively on the graft bed, graft margin, and donor skin. It was possible to observe a non-linear and negative relationship between the variables for all perfusion measurements. A curve estimation approach explored the non-linear relationships between the different perfusion measurements and the necrosis extension. The logarithmic model fitted better than the others (inverse, exponential, and polynomial), so the variable “CVC” underwent logarithmic conversion for the subsequent linear correlation and linear regression analyses. The following perfusion results will be presented as CVC’s natural logarithm [Ln (CVC)]. 

On the scalp, we observed a strong correlation between graft bed perfusion and necrosis extension (r = −0.83, *p* < 0.001). The graft margin and donor skin perfusions did not correlate with necrosis extension for grafts in this location (r = −0.31, *p* = 0.15 and r = −0.43, *p* = 0.10, respectively).

On the lower limb, graft bed and graft margin perfusions were very strongly (r = −0.94, *p* < 0.001) and moderately (r = −0.57, *p* = 0.01) correlated with necrosis extension, respectively. Nevertheless, the correlation between the graft bed and graft margin perfusions was moderate (r = −0.53, *p* = 0.02). There was no correlation between donor skin perfusion and necrosis extension (r = −0.24, *p* = 0.41).

Fisher’s z-test was performed to compare the correlation between graft bed perfusion and necrosis extension on the scalp and lower limb. The difference between the correlations was statistically significant, with the correlation being stronger in the lower limb (z = 1.8, *p* = 0.03).

Table 3 and Table 4 describe the univariate and multivariate linear regression models of necrosis based on the previously identified significant scalp and lower limb perfusion factors. 

For skin grafts on the scalp, graft bed perfusion explained 68% of the variation in necrosis extension. Based on graft bed perfusion alone, the predictive model of necrosis extension on day 28 can be represented by the formula “necrosis extension = 0.07 − 0.48 × Ln (CVC graft bed)”.

On the lower limb, the models based on intraoperative graft bed and marginal skin perfusions were statistically significant, explaining 89% and 29% of the extent of necrosis, respectively. However, the multivariate model, including the two intraoperative measurements, did not fit better than the one with bed perfusion alone. Thus, the best linear regression model for predicting the graft necrosis extension on the lower limb is based on graft bed perfusion and can be expressed as “necrosis extension = 0.16 − 0.40 × Ln (CVC graft bed)”.

For every one-unit increase in bed perfusion [in Ln (CVC)], the necrosis extension decreased by 40 percentage points on the lower limb and 48 percentage points on the scalp. Still, there was an intersection of the 95% confidence intervals of the beta coefficients for the models based on graft bed perfusion on the scalp (−0.63 to −0.33) and lower limb (−0.46 to −0.33). Consequently, the effect of graft bed perfusion on necrosis extension did not differ between locations.

## 4. Discussion

This work was conceived to evaluate the role of perfusion measured on the graft bed, graft margin, and donor skin in the skin graft necrosis extension, either on the scalp or lower limb.

We observed that graft bed perfusion very strongly correlated with necrosis extension, explaining more than two-thirds of its variation on the scalp and almost 90% of tis variation on the lower limb. Despite a similar baseline graft bed perfusion in both locations, the correlation with necrosis extension and the ability to predict necrosis based solely on graft bed perfusion were stronger for the lower limb than for the scalp. Still, the mean reduction in necrosis extension with increasing graft bed perfusion was similar across locations, despite differences in the total variability in necrosis extent attributed to anatomical location (i.e., different R^2^ values).

The graft margin perfusion only correlated with necrosis on the lower limb, but it did not outperform the graft bed perfusion in predicting graft necrosis in any location.

We have observed higher perfusion in the clavicular region (donor skin for scalp) than in the anterior tight (donor skin for lower limb), which is probably due to the expected differences in native vascular density. This perfusion did not correlate with graft necrosis extension in either location. 

Nonetheless, we cannot deny the importance of the quality of donor skin or graft margin on skin graft viability. Based on our evidence, their contribution to graft survival might be explained by factors other than their perfusion status.

The quality of the skin surrounding the graft is expected to be relevant to graft viability by guaranteeing the anchorage of the grafts to the edges of the surgical wound and possibly contributing to nutrition through soaking in the first hours of ischaemia. The bridging phenomenon (i.e., the revascularisation from the wound edges instead of the wound bed) has been described as crucial for skin graft viability in poorly vascularised graft beds [24]. This concept has been recently questioned, since Abdelhakim et al. observed no reperfusion from the wound margin in murine models of skin grafting [25]. This might explain the lack of correlation between wound margin perfusion and necrosis extension on the scalp. In the lower limb, the margin perfusion’s ability to predict necrosis extension probably derives from its (moderate) relationship with wound bed perfusion.

The density of hair follicles in the donor skin can contribute to the viability of the graft by mechanisms not directly related to perfusion since they are a source of stem cells that can migrate to the bed and influence the healing process [26]. Likewise, if a full-thickness graft includes subcutaneous fat, there is potential for residual adipose-derived stem cells to remain in the tissue. These cells could theoretically contribute to the graft’s regenerative capacity or influence wound healing through paracrine signalling mechanisms, as they secrete cytokines, growth factors, and extracellular vesicles with immunomodulatory effects [27]. 

The two analysed groups showed no significant difference regarding the conditions leading to microcirculation dysfunction or an increased risk of graft failure. Moreover, they shared the same graft bed perfusion. So, we must acknowledge that surgical-related factors we could not address might contribute to the differences observed in the models’ predictive capacity. The dense scalp vascular supply makes excisions in this location more prone to bleeding, especially from the borders, compared to lower limb excisions [28]. Consequently, a more intense cauterisation of the wound might temporarily decrease the perfusion measurements in this location. The accumulation of blood depots between the bed and the graft might also impair graft reperfusion and increase the risk of necrosis (even though grafts with evidence of haematoma have been excluded). We also wonder if the high correlation coefficient (r) and determination coefficient (R^2^) observed on the lower limb for graft bed perfusion might derive from the exclusive usage of split-thickness skin grafts in this location. These grafts are expected to tolerate the early ischemic period of revascularisation better due to their inherent lower metabolic activity (because of the lower density of adnexal structures) [29]. Consequently, their viability might more accurately reflect the perfusion status of the wound bed than split-thickness skin grafts (whose thickness could not be controlled).

Despite the consensus in the literature that the better the graft bed is perfused, the better the graft viability, so far, it has not been quantified how much of this viability depends on the perfusion of the wound bed [30]. Not only are lower limb and scalp grafts reperfused similarly (as observed in our previous work), but their necrosis extent also decreases similarly in response to increases in wound bed perfusion [22].

Our data might have direct clinical implications by identifying predictive models of necrosis on the scalp and lower limb based on intraoperative objective perfusion measurements with high accuracy. If LSCI is employed intraoperatively, we might be able to make decisions about immediate or delayed graft closure, according to the expected necrosis extension. The same rationale can be used after preconditioning strategies of the wound bed to determine the appropriate time for ultimate graft closure based on the predicted necrosis extension. 

Between 2020 and 2044, the incidence of non-melanoma skin cancer is expected to increase by 1.5 times, driven by longer life expectancy, greater UV exposure, and improved diagnostic accuracy [31]. In Europe, melanoma cases are projected to rise by 50% between 2020 and 2040 [32]. This anticipated increase highlights the growing demand for skin grafts, a relatively simple reconstructive option requiring less surgical expertise than flaps. Therefore, it is essential to develop reconstructive strategies that improve cosmetic and functional results. In our opinion, these should include predicting skin graft necrosis extension, among other aspects. 

### Limitations

Our predictive models must be interpreted according to the study’s limitations. This was a single-centre cohort study of elderly patients with cardiovascular comorbidities, in whom some surgical-related factors could not be assessed. Despite being a typical population of ambulatory-treated skin cancer patients, younger subjects submitted to skin grafts under general anaesthesia in a non-oncologic setting might have different results. If different non-invasive skin imaging techniques (with different temporal and spatial relationships and skin penetration) are employed, the regression models might diverge from ours.

## 5. Conclusions

In conclusion, perfusion plays a significant role in skin graft survival, with the graft bed perfusion status being more relevant than the margin or donor skin, on either scalp or lower limb. Nevertheless, including perfusion assessment in reconstructive algorithms with skin grafts in these locations can potentially improve the success of this procedure.

## Figures and Tables

**Table 1:** Characteristics of participants, the defect size, and the graft necrosis extension according to the anatomic location of the skin graft.

Participant Characteristics	Scalp *n* = 22	Lower Limb *n* = 20	*p*
Age (years)	79 (61–96, IQR 16)	82 (68–94, IQR 10)	0.58
Women	4 (18%)	14 (70%)	0.001
Hypertension	17 (77%)	19 (95%)	0.18
Dyslipidaemia	12 (55%)	13 (65%)	0.54
Diabetes mellitus	5 (23%)	4 (22%)	1.00
Smokers	1 (5%)	0 (0%)	1.00
Under antiplatelets	4 (18%)	4 (20%)	1.00
Under anticoagulant	5 (23%)	4 (20%)	1.00
Defect characteristics			
Defect area (cm^2^)	11 (2.2–47, IQR 7.2)	14 (5.5–31, IQR 13)	0.09
Graft characteristics			
Graft necrosis (%)	12 (0–84, IQR 14)	31 (2–81, IQR 34)	0.06

Note: Values are presented as the median, maximum, minimum, and interquartile range (IQR) for continuous variables and as absolute and relative frequency for categorical variables.

**Table 2:** Comparison of intraoperative perfusion values between the scalp and the lower limb.

CVC Measurement	Scalp	Lower Limb	*p*
Donor skin	1.4 (0.8–2.2) ± 0.32	0.7 (0.4–1.1) ± 0.22	<0.001
Graft bed	0.83 (0.27–1.3) ± 0.28	0.79 (0.25–1.6) ± 0.41	0.69
Graft margin	1.1 (0.40–2.3) ± 0.44	0.51 (0.20–0.90) ± 0.25	<0.001

Note: Perfusion was measured as cutaneous vascular conductance (CVC) in APU/mmHg. Mean, minimum, maximum, and standard deviation values are shown.

**Table 3:** Univariate linear regression model of necrosis extension based on the perfusion of the graft bed on the scalp.

Scalp	*b*	*p*	CI _95%_ *b*		Model Summary
Univariate model					
*Constant*	0.07	0.04	0.01	1.1	R^2^ = 0.68 F (1, 20) = 43 *p* < 0.001
Ln (CVC graft bed)	−0.48	<0.001	−0.63	−0.33	

Note: ***b***, beta coefficient; CI, confidence interval; CVC, cutaneous vascular conductance; Ln, natural logarithm.

**Table 4:** Univariate and multivariate linear regression models of necrosis extension based on the perfusion of the graft bed on the lower limb.

Lower Limb	*b*	*p*	CI _95%_ *b*		Model Summary
Univariate model					
*Constant*	0.16	<0.001	0.11	0.20	R^2^ = 0.89 F (1, 18) = 150 *p* <0.001
Ln (CVC graft bed)	−0.40	<0.001	−0.46	−0.33	
*Constant*	0.11	0.17	−0.05	0.28	R^2^ = 0.29 F (1, 18) = 8.6 *p* = 0.01
Ln (CVC margin)	−0.24	0.01	−0.42	−0.07	
Multivariate model					
*Constant*	0.15	<0.001	−0.08	−0.22	R^2^ = 0.88 F (2, 19) = 72 *p* < 0.001
Ln (CVC graft bed)	−0.39	<0.001	−0.42	−0.30	
Ln (CVC margin)	−0.02	0.07	−0.10	0.07	

Note: ***b***, beta coefficient; CI, confidence interval; CVC, cutaneous vascular conductance; Ln, natural logarithm.

## Data Availability

The data supporting this study’s findings are available from the corresponding author upon reasonable request.
